# Asparagine endopeptidase (AEP) inhibitor formulation via zein-based nanoparticle improves the therapeutic efficacy toward Alzheimer's disease

**DOI:** 10.1016/j.neurot.2025.e00718

**Published:** 2025-08-14

**Authors:** Xin Meng, Mengmeng Wang, Menghan Yang, Guangxing Wang, Zhenlei Zhao, Zhongyun Xie, Bowei Li, Zhengjiang Qian, Seong Su Kang, Wenhua Zheng, Keqiang Ye

**Affiliations:** a Department of Pharmaceutical Sciences, Faculty of Health Sciences, University of Macau, Taipa, 999078, Macao Special Administrative Region of China; b Faculty of Life and Health Sciences, Shenzhen University of Advanced Technology (SUAT), Shenzhen, Guangdong 518055, China; c Brain Cognition and Brain Disease Institute (BCBDI), Shenzhen Institutes of Advanced Technology (SIAT), Chinese Academy of Sciences, Shenzhen, 518055, Guangdong, China; d University of Chinese Academy of Sciences (UCAS), Shenzhen Institutes of Advanced Technology (SIAT), Chinese Academy of Sciences, China; e Department of Laboratory Medicine, Sichuan Provincial People's Hospital, University of Electronic Science and Technology of China, Chengdu, Sichuan 610072, China; f Zhejiang Key Laboratory of Geriatrics and Geriatrics Institute of Zhejiang Province, Zhejiang Hospital, 310030, Hangzhou, China; g Department of Pathology and Laboratory Medicine, Emory University School of Medicine, Atlanta, GA 30322, USA

**Keywords:** Nanoparticles, AEP inhibitor, Formulation, Pharmacokinetics, Alzheimer's disease

## Abstract

Asparagine endopeptidase (AEP) plays a critical role in Alzheimer's disease (AD) by cleaving amyloid precursor protein (APP) at N585 and tau protein at N368. Genetic deletion or pharmacological inhibition of AEP using compound 11a ameliorates AD pathology in murine models. To improve the therapeutic potential of 11a, we synthesized structural analogs and developed a zein-based nanoparticle delivery system to enhance pharmacokinetics. Structural modification, specifically isopropyl substitution of the N-methyl group in 11a, markedly improved blood-brain barrier permeability. The lead compound, 11a-isopropyl, formulated in zein nanoparticles, exhibited superior oral bioavailability and brain exposure. *In vivo* pharmacodynamic/pharmacokinetic (PK/PD) analyses confirmed dose-dependent AEP inhibition and enhanced substrate stabilization, with the nanoparticle formulation further increasing efficacy. One-month oral administration in 3xTg AD mice demonstrated that 11a-isopropyl, particularly in nanoparticle form, significantly reduced Aβ and tau pathology and improved cognitive performance. These findings indicate that zein-based nanoparticles enhance AEP inhibitor delivery and therapeutic efficacy in AD.

## Introduction

Alzheimer's disease (AD) is a progressive, age-related neurodegenerative disorder and the most prevalent form of dementia. It is characterized by memory loss, neuropsychiatric symptoms, and a declining ability to perform daily activities. Its pathological hallmarks include the accumulation of extracellular senile plaques composed primarily of aggregated β-amyloid peptide (Aβ) and intracellular neurofibrillary tangles (NFTs) formed by hyperphosphorylated and truncated tau proteins [1,2]. Emerging evidence highlights chronic neuroinflammation driven by sustained glial activation and widespread neuronal apoptosis as key pathological contributors to AD progression.

Asparagine endopeptidase (AEP), also known as legumain or δ-secretase, is a lysosomal cysteine protease activated under acidic conditions and cleaves substrates specifically at the C-terminus of asparagine residues [3,4]. Under ischemic or excitotoxic conditions, AEP is proteolytically processed from its inactive 55 ​kDa form to an active 37 ​kDa fragment. This active form cleaves the nuclear protein SET at residue N175, triggering neuronal cell death [5]. Under physiological conditions, AEP activity is tightly regulated within lysosomes by cystatin C. However, in AD patients, reduced cystatin C levels in cerebrospinal fluid (CSF) and a lowered brain pH result in aberrant AEP activation [[6], [7], [8]]. Cystatin C also directly binds to soluble Aβ, inhibiting its oligomerization and exerting neuroprotective effects [9].

Our previous studies have shown that AEP contributes significantly to Aβ production by cleaving amyloid precursor protein (APP). Both AEP expression and APP cleavage by AEP are upregulated with age in mouse models and human AD brains. Specifically, AEP cleaves APP at residues N373 and N585, producing two pathogenic fragments. The C-terminal fragment (APP 586–695) serves as an optimal substrate for β- and γ-secretases, thereby enhancing Aβ generation, while the N-terminal fragment (APP 1–373) is directly neurotoxic. These findings classify AEP as a δ-secretase with secretase-like activity. Genetic deletion of AEP in APP/PS1 and 5XFAD transgenic mice attenuates AD-associated pathologies, including synaptic loss, Aβ deposition, amyloid plaque burden, and cognitive impairment [10]. In addition to Aβ pathology, NFTs composed of truncated and hyperphosphorylated tau are another hallmark of AD. Tau fragmentation disrupts microtubule assembly, promoting its aggregation into neurotoxicity [11]. AEP cleaves Tau at residue N368, generating aggregation-prone fragments that promote hyperphosphorylation and NFT formation [[12], [13], [14], [15], [16], [17]]. AEP cleaves tau at residues N255 and N368 independently of caspases or calpains. The resulting fragment, Tau (1–368), is highly neurotoxic, promotes tau hyperphosphorylation and aggregation, and is abundant in AD brains but scarce in controls. Genetic ablation of AEP in Tau P301S transgenic mice markedly reduces NFT pathology and improves memory function [18]. AEP is the only known protease that cleaves both APP and tau, directly driving AD pathogenesis. Furthermore, AEP interacts with β-secretase (BACE1), cleaving it at residue N294 in an age-dependent manner, which enhances BACE1 activity and Aβ production. The truncated BACE1 (1−294) fragment enhances Aβ generation and accelerates cognitive decline in APP/PS1 mice [19]. Additionally, the Tau (1–368) fragment activates STAT1, a transcription factor that upregulates BACE1 expression, creating a feedforward loop that exacerbates Aβ pathology [20]. This crosstalk between δ- and β-secretases exacerbates AD pathology. Notably, pharmacological inhibition of AEP with compound #11, a small-molecule inhibitor, reduces Tau and APP cleavage, alleviating AD-related pathology and cognitive deficits in Tau P301S and 5XFAD mice [21].

To overcome poor drug absorption, nanoparticle (NP)-based delivery systems have emerged as promising platforms to enhance the pharmacokinetics of therapeutic compounds [[22], [23], [24]]. These systems improve oral bioavailability by stabilizing compounds in the gastrointestinal tract and facilitating their absorption. Surface-modified NPs can also cross the blood-brain barrier (BBB), offering potential for central nervous system drug delivery in AD [25,26].

Zein, a hydrophobic protein derived from corn, is soluble in ethanol and organic solvents. It is classified as “generally recognized as safe” (GRAS) and approved by the FDA for use as an inactive pharmaceutical ingredient [27], zein's hydrophobicity, biodegradability, and biocompatibility make it useful for food coating, packaging, pill coatings [28], drug delivery, and tissue engineering [29]. It can self-assemble into nanoparticles with various morphologies depending on solvent and processing conditions [30]. Its rich functional groups (hydroxyl, amino, and carboxyl) allow chemical conjugation via crosslinking agents. However, zein nanoparticles tend to aggregate under high salt concentrations, heat, or pH values near their isoelectric point, limiting their stability and utility [31]. Lactoferrin (LF), an iron-binding glycoprotein, has been shown to stabilize NPs effectively [32,33]. LF-targeted NPs benefit from enhanced uptake via transferrin receptors expressed on intestinal epithelial cells [34]. Recently, we developed zein/LF-based NPs to improve the oral bioavailability and efficacy of a CF3CN-derived TrkB receptor agonist in AD models [35]. In this study, we applied the zein/LF delivery system to enhance brain exposure of the compound 11a-isopropyl, a potent AEP inhibitor. We synthesized several derivatives of 11a with dose-dependent potency comparable to the parent compound and evaluated their pharmacokinetic and pharmacodynamic (PK/PD) profiles, as well as therapeutic efficacy, in an AD mouse model.

## Results

### Optimization of AEP inhibitor #11a via medicinal chemistry

Metabolite analysis showed that the N-methyl group on compound 11a is readily metabolized by liver microsomes, resulting in a reduced *in vivo* half-life [36]. To optimize metabolic stability and increase lipophilicity, we synthesized several derivatives, including 11a-pentyl and 11a-isopropyl as representative compounds (Fig. 1A). *In vitro* AEP enzymatic inhibition assays showed that cyclopentylation or isopropylation decreased the potency compared to #11a. The IC_50_ increased from 6.8 ​nM (for 11a) to 14.7 and 19.2 ​nM, respectively. Similarly, in primary neurons, the IC_50_ for AEP inhibition increased from 45 ​nM (11a) to 55 ​nM (pentyl) and 63 ​nM (isopropyl) (Fig. 1B&C). These results suggest that enhancing the hydrophobicity of the primary amine group slightly compromises antagonistic potency. To determine whether 11a-isopropyl effectively blocks Aβ42-induced AEP activation, we performed a titration assay. 11a-isopropyl suppressed AEP activation in a dose-dependent manner, with downstream inhibition of APP N585 and Tau N368 proteolytic fragments. The ratios of Tau N368/Tau and APP N585/APP aligned with the cellular AEP IC_50_ value (Fig. 1D&E), confirming membrane permeability and functional AEP inhibition in neurons. To evaluate druggability, we assessed the compounds’ *in vitro* ADMET profiles. Cyclopentylation and isopropylation both substantially reduced the water solubility compared to 11a. Notably, cyclopentylation destabilized microsomal stability in both human and mouse liver microsomes, whereas isopropylation improved stability in human microsomes, suggesting species-specific differences in metabolism. Both modifications, however, led to reduced hepatocyte stability in human and mouse models. In contrast, plasma stability remained high across all compounds. Plasma protein binding was significantly reduced in both derivatives compared to 11a. Brain penetration, assessed via PAMPA-BBB assay, was notably improved with cyclopentylation and isopropylation. However, both derivatives exhibited increased Papp values in the MDR1-MDCKII assay, indicating they are likely P-glycoprotein substrates and subject to efflux (Table S1). Further evaluation via Caco-2-BCRP assays demonstrated that 11a-isopropyl is not a significant substrate of BCRP. Although the baseline efflux ratio (ER ​= ​2.95) exceeded the threshold for potential transporter involvement (ER ​≥ ​2.0), co-administration with the selective BCRP inhibitor Ko143 reduced ER to only 2.27 (23 ​% suppression). This level of inhibition did not meet the criterion for BCRP substrate classification (requiring >50 ​% ER reduction; Table S2). Interestingly, Caco-2 permeability results showed decreased absorption for 11a-isopropyl and enhanced absorption for 11a-pentyl, relative to 11a. Lastly, hERG inhibition assays demonstrated that IC_50_ values for K^+^ channel blocking were significantly higher than the concentrations required for AEP inhibition, suggesting a low risk of cardiotoxicity (Table S1).

### Characterization of zein/lactoferrin nanoparticles containing 11a-isopropyl (NP-11a-isopropyl)

To enhance the *in vivo* PK profiles of 11a-isopropyl, we prepared zein NPs using the antisolvent co-precipitation method, varying the mass ratios between the cargo (11a-isopropyl) and zein. The NPs were characterized by dynamic light scattering (DLS) to determine the particle sizes, zeta potential, and polydispersity index (PDI). We identified that the zein:11a-isopropyl mass ratio of 10:1 yielded NPs with the optimal characteristics (Fig. S1A–D). However, zein-based NPs are prone to aggregation and precipitation. To address this, we further modified the NPs by incorporating lactoferrin (LF), an iron-binding glycoprotein known to stabilize NPs [44,45]. To obtain the best mass ratio between zein and LF, we tested different zein:LF mass ratios and found that a 1:1 ratio produced the most stable cargo-loaded zein/LF composite NPs (11a-isopropyl/zein/LF) (Fig. S1E–H). To further stabilize the 11a-isopropyl/zein/LF NPs, we employed the Maillard reaction to glycosylate LF proteins using dextran, resulting in 11a-isopropyl/zein/DLF NPs (Fig. 2A). We then quantitatively compared the NPs of 11a-isopropyl/zein, 11a-isopropyl/zein/LF, and 11a-isopropyl/zein/DLF. Key parameters, including particle size, zeta potential, PDI, turbidity, encapsulation efficiency (EE%), and loading capacity (LC%), were characterized (Fig. 2B−G). Analysis of EE and LC revealed that glycosylation of LF reduced both EE and LC in zein/LF NPs (Fig. 2F and G). Storage stability analysis indicated that zein/DLF NPs were significantly less stable than zein/LF NPs (Fig S2A&B). A summary of EE (%), LC (%), particle size, and PDI for these NPs is provided in Fig. S2C. Size distribution by intensity and zeta potential distribution, as measured by DLS, are shown in Fig. S2D&E. Both scanning electron microscopy (SEM) and transmission electron microscopy (TEM) confirmed that the NPs were uniformly spherical (Fig. 2H).

**Fig. 1:**
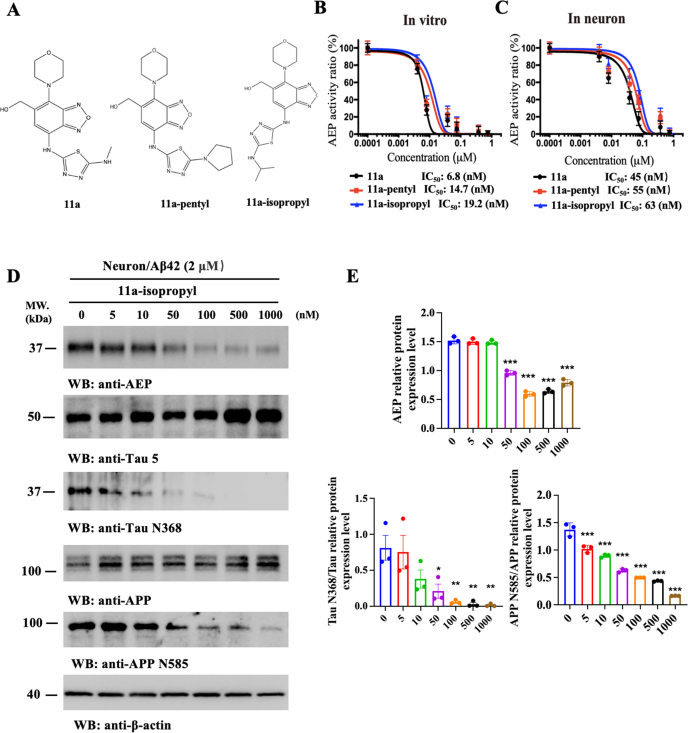
**Validation of AEP inhibitors *in vitro*.** (A) Chemical structures of the 3 candidates. (B) IC_50_ values of the compounds toward recombinant AEP enzyme. (C) IC_50_ values of the compounds in cultured primary neurons. (D) Immunoblotting analysis to verify the effects of AEP inhibitors on AEP and AEP cleavage substrates in Aβ-induced primary neuronal models *in vitro*. (E) Quantification of (D) (n ​= ​3 biological replicates; data are shown as means ​± ​S.D.).

**Fig. 2:**
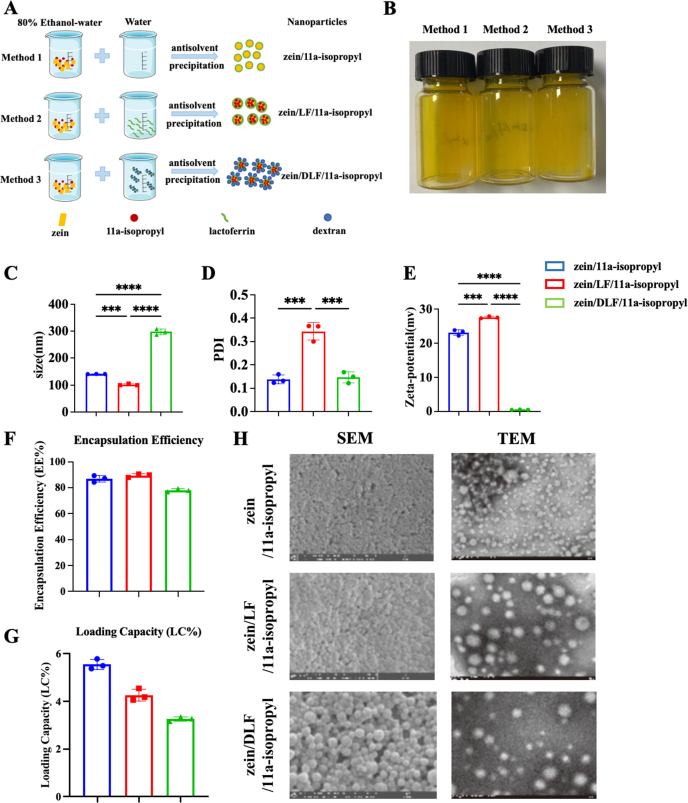
**Characterization of Zein/LF NPs containing 11a-isopropyl.** (A) Structural illustration of the formation mechanism of zein/11a-isopropyl, zein/LF/11a-isopropyl, and zein/DLF/11a-isopropyl NPs. (B–E) NP drug solution, particle size, zeta potential, and PDI of zein-based NPs were measured. (F–G) EE (%) and drug LC (%) of 11a-isopropyl-loaded zein-based NPs were determined (n ​= ​3 biological replicates; data are shown as means ​± ​S.D.). (H) SEM images of zein-based NPs taken at 50,000 ​× ​magnification. Scale bar ​= ​1 ​μm. TEM images of zein-based NPs. Scale bar ​= ​500 ​nm.

### *In vivo* pharmacokinetic analysis of NP-11a-isopropyl

To evaluate the *in vivo* PK profiling features of 11a-isopropyl before and after NP formulation, we resuspended 11a-isopropyl and the lyophilized zein-based NPs and quantitatively compared their delivery into the circulation system and the brain after oral administration. We conducted the *in vivo* PK study using 2-month-old ICR mice. Oral administration of 11a-isopropyl (5 ​mg/kg) showed that the half-life (t_1/2_) in the plasma was approximately 0.73 ​h, whereas i.v. injection (2 ​mg/kg) exhibited 0.89 ​h in the plasma. The area under the curve (AUC) values were 2412.38 ​ng/mL·h for i.v. and 4050.46 ​ng/mL·h for oral administration (P.O.), with an oral bioavailability of approximately 67.16 ​%. Clearance rates (CL: L/h/kg) increased from 0.83 for 11a-isopropyl to 1.24 for NP-#11a-isopropyl. The maximal plasma concentrations (C_max_) were 5158.15 ​ng/mL (i.v.) and 4773.85 ​ng/mL (P.O.), respectively. Notably, the C_max_ concentrations of 11a-isopropyl in the brain were 306.77 ​ng/mL (i.v.) and 321.25 ​ng/mL (P.O.), with brain half-lives of ∼2 ​h and 2.4 ​h, respectively. The brain AUC values were approximately 921.71 ​ng/mL·h (i.v.) and 385.36 ​ng/mL ​h ​(P.O.) (Fig. 3A–F). Compared to 11a, the C_max_, and AUC values for 11a-isopropyl in plasma and brain were significantly enhanced after oral administration, while the brain half-life (t_1/2_) remained comparable [37]. These results demonstrate that isopropylation substantially increases 11a's plasma and brain exposure.

**Fig. 3:**
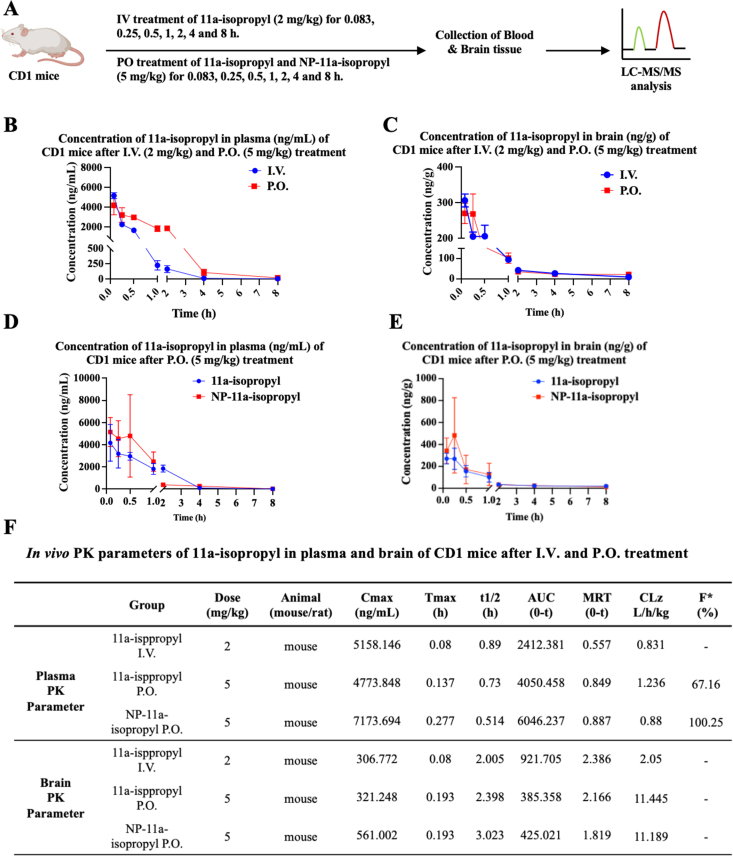
***In vivo* pharmacokinetics (PK) profiles.** 11a-isopropyl and NP-11a-isopropyl were administered via intravenous (I.V.) injection or oral administration (P.O.) in CD1 mice. (A) Diagram showing experimental scheme for *in vivo* PK profile analysis of 11a-isopropyl and NP-11a-isopropyl. A total of 63 CD1 mice were equally divided into three groups, which were subjected to 11a-isopropyl and NP-11a-isopropyl treatment by I.V. (2 ​mg/kg) and P.O. (5 ​mg/kg) treatment, respectively. Blood and brain tissue were collected from three mice at each indicated time point, and the concentrations of 11a-isopropyl were determined by LC-MS/MS for PK parameters analysis. (B) and (C) Concentrations of 11a-isopropyl in the plasma (ng/mL) and brain (ng/g) of CD1 mice after I.V. and P.O. treatment with 11a-isopropyl at each indicated time point. (D) and (E) Concentrations of 11a-isopropyl in the plasma (ng/mL) and brain (ng/g) after P.O. treatment with 11a-isopropyl and NP-11a-isopropyl in CD1 mice. (F) *In vivo* plasma and brain PK parameters of 11a-isopropyl and NP-11a-isopropyl in CD1 mice by I.V. and P.O. treatment.

When comparing 11a-isopropyl to its NP-formulated counterpart, the C_max_ values increased from 4773.85 ​ng/mL to 7173.69 ​ng/mL in plasma and from 321.25 ​ng/mL to 561.00 ​ng/mL in the brain following zein-LF NP encapsulation. Oral bioavailability improved from 67.16 ​% to 100.25 ​% (Fig. 3F). In the brain, the AUC values after oral administration increased from 385.36 ​ng/mL·h to 425.02 ​ng/mL·h, and the t_1/2_ extended from 2.4 ​h to 3.02 ​h after NP encapsulation. These findings indicate that the zein/LF NP formulation significantly enhances 11a-isopropyl brain exposure and prolongs its half-life in the brain.

### *In vivo* PK/PD correlation of oral administrated NP-11a-isopropyl in 3xTg mice

To assess whether NP-11a-isopropyl administration efficiently blocks AEP protease activity, we employed 6-month-old 3xTg AD mice and analyzed AEP enzymatic activity and the fragmentation of its downstream targets in the brain lysates 2 ​h after oral gavage of 7.5 ​mg/kg and 15 ​mg/kg of drug (Fig. 4A). The plasma and brain concentrations of the 11a-isopropyl compound were quantitatively analyzed by LC/MS/MS. Quantification revealed that AEP enzymatic activities in the brain were substantially inhibited by 11a, 11a-isopropyl, and its NP formulation in a dose-dependent manner, which tightly correlated with AEP inhibitory effects. As expected, the zein/LF formulation significantly elevated 11a-isopropyl levels in the brains of 3xTg mice, increasing from 14.411 to 18.964 ​ng/mL for the 7.5 ​mg/kg dose and from 44.658 to 56.109 ​ng/mL for the 15 ​mg/kg dose (Fig. 4B and C). Immunoblotting analysis of hippocampal tissues showed that active AEP band intensities were reduced by 11a and further suppressed by 11a-isopropyl. Consequently, the levels of APP N585 and Tau N368, two fragments generated by AEP cleavage of APP and Tau, were decreased by these inhibitors, consistent with the observed AEP inhibitory activities (Fig. 4D). Additionally, immunoblotting demonstrated that the NP formulation enhanced 11a-isopropyl inhibitory activity by blocking AEP activation and the proteolytic fragmentation of downstream substrates in a concentration-dependent manner (Fig. 4E). The immunoblotting results were quantitatively analyzed (Fig. 4F&G). To exclude nonspecific effects of the nanocarrier, a zein/LF nanoparticle-only control group was included. No significant changes in AEP activity or substrate cleavage were observed compared to the vehicle, confirming pharmacological effects are compound-dependent (Fig. 4E). Hence, the acute *in vivo* PK/PD studies support that 11a-isopropyl targets AEP protease in the brains of 3xTg mice, blocking APP N585 and Tau N368 proteolytic fragmentation, and that the zein/LF formulation significantly enhances 11a-isopropyl inhibitory potency.

**Fig. 4:**
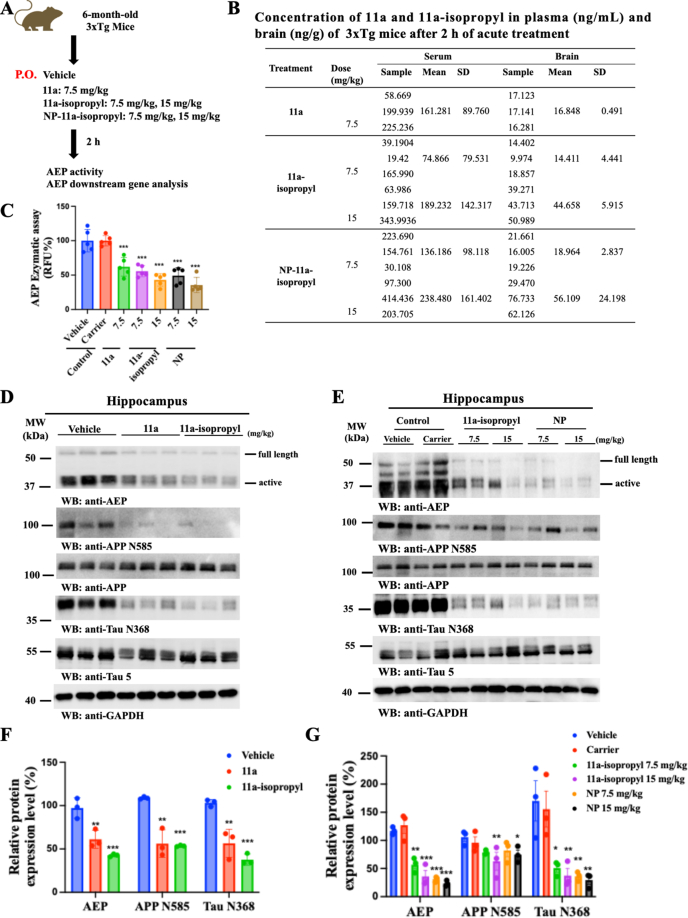
***In vivo* PD/PK analysis.** Acute treatment with 11a-isopropyl and NP-11a-isopropyl inhibits the activity of AEP and its downstream targets in 3xTg mice. (A) Diagram showing experiment of acute 11a, 11a-isopropyl, and NP-11a-isopropyl treatment in 3xTg mice. The mice were treated with 11a-isopropyl and NP-11a-isopropyl at different concentrations (7.5 and 15 ​mg/kg) by P.O. for 2 ​h. The blood and brain tissue were collected from three mice treated with each indicated concentration of 11a, and the 11a-isopropyl level, AEP activity, and the downstream targets of AEP were analyzed. (B) Concentrations of 11a and 11a-isopropyl in the plasma (ng/mL) and brain (ng/g) of 3xTg mice with acute treatment of 11a and 11a-isopropyl. (C) AEP enzymatic activity in the brain tissue of 3xTg mice with acute treatment of 11a, 11a-isopropyl, and NP-11a-isopropyl at different concentrations (n ​= ​5 biological replicates, ∗∗∗*p* ​< ​0.001, compared with vehicle). (D) Representative Western blot images showing the effects of 11a and 11a-isopropyl (7.5 ​mg/kg) treatment on the expression of AEP and its downstream targets in 3xTg mice. (E) Representative Western blot images showing the effects of different doses of 11a-isopropyl and NP-11a-isopropyl (7.5 ​mg/kg and 15 ​mg/kg) treatment on the expression of AEP and its downstream targets in 3xTg mice. (F) and (G) Relative quantification of protein levels of (D) and (E), respectively. (n ​= ​3 biological replicates; ∗*p* ​< ​0.05, ∗∗*p* ​< ​0.01, and ∗∗∗*p* ​< ​0.001, compared with vehicle).

### Chronic treatment with NP-11a-isopropyl inhibits AEP biological function in the brain of 3xTg mice

To investigate the therapeutic efficacy of 11a-isopropyl and its zein/LF-formulated version, we selected 5-month-old 3xTg mice and administered 7.5 ​mg/kg of the compound daily via oral gavage for one month. At the end of the treatment, immunoblotting analysis of the hippocampal tissues revealed that AEP activation, APP N585, and Tau N368 fragmentation were gradually reduced from 11a to 11a-isopropyl to NP-11a-isopropyl (Fig. 5A–C). These results support the conclusion that isopropylation enhances the inhibitory potency of 11a and that zein/LF formulation further amplifies this activity. Immunofluorescent (IF) co-staining of hippocampal sections was performed using antibodies against active AEP and C586-695, which specifically recognizes the AEP-cleaved C-terminus of APP, containing Aβ peptide that is more prone to be further shredded by BACE1 into C99 [10]. Again, IF analysis demonstrated that active AEP abundance and C586 signals were all markedly diminished by these inhibitors (Fig. 5D&E). Consistent with these findings, the AEP enzymatic assay further validated this observation (Fig. 5F).

**Fig. 5:**
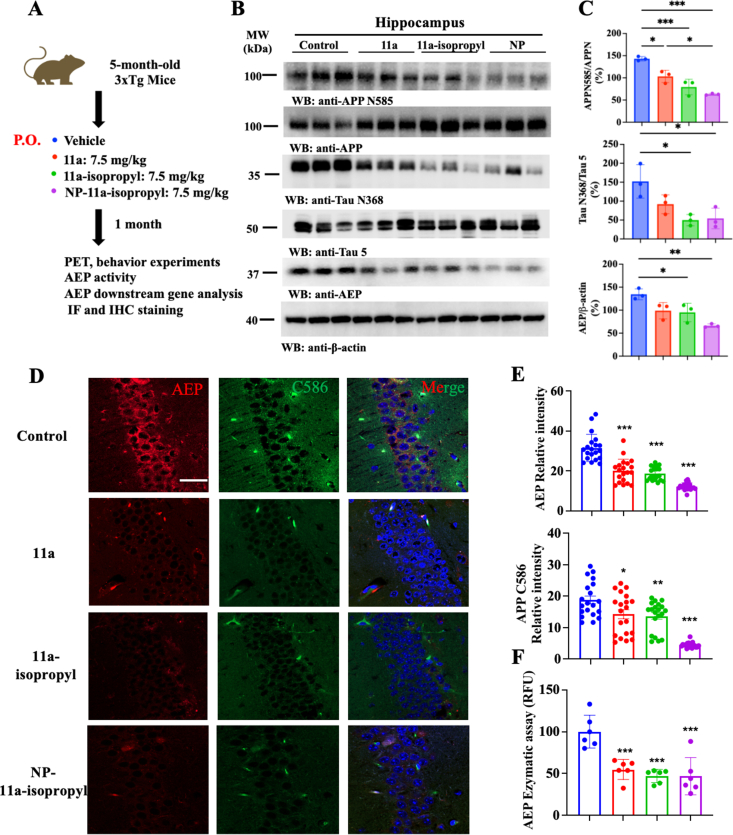
**Chronic treatment with 11a-isopropyl and NP-11a-isopropyl inhibits the activity of AEP and its downstream targets in 3xTg mice.** (A) Diagram showing experiment scheme of chronic 11a, 11a-isopropyl, and NP-11a-isopropyl treatment in 3xTg mice. The mice were treated with 11a, 11a-isopropyl, and NP-11a-isopropyl (7.5 ​mg/kg) by P.O. for one month. (B) Representative Western blot images showing the effects of 11a, 11a-isopropyl, and NP-11a-isopropyl treatment on the expression of AEP and its downstream targets in 3xTg mice. (C) Relative quantification of protein levels in (B). (n ​= ​3 biological replicates; ∗*p* ​< ​0.05, ∗∗*p* ​< ​0.01, and ∗∗∗*p* ​< ​0.001, compared with vehicle). (D) Immunofluorescence staining of AEP and APP C586 in the hippocampus of 3xTg mice with chronic 11a, 11a-isopropyl, and NP-11a-isopropyl treatment. Scale bar, 20 ​μm. (E) Relative quantification of AEP and APP C586 fluorescence intensity in (D) (20 positive cells from three mice for statistics, ∗*p* ​< ​0.05, ∗∗*p* ​< ​0.01, and ∗∗∗*p* ​< ​0.001, compared with vehicle). (F) AEP enzymatic activity (%) in the brain tissue of 3xTg mice with chronic treatment of 11a, 11a-isopropyl, and NP-11a-isopropyl (n ​= ​6 biological replicates, ∗∗∗*p* ​< ​0.001, compared with vehicle).

### Chronic treatment with NP-11a-isopropyl alleviates AD pathology and improves cognitive functions

**Fig. 6:**
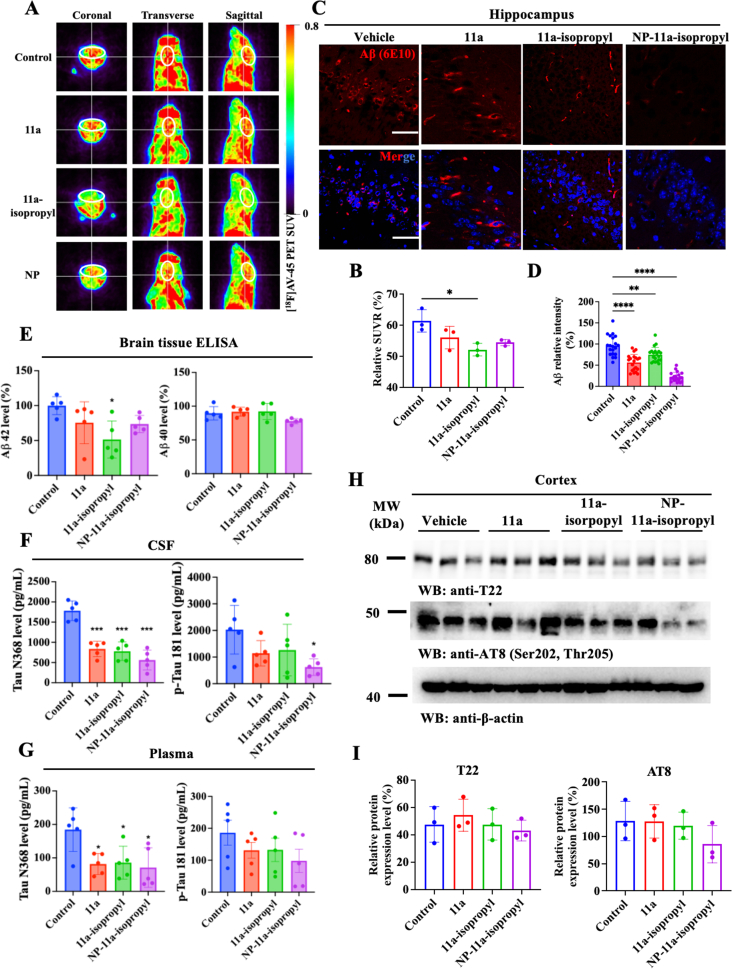
**Chronic treatment with 11a-isopropyl and NP-11a-isopropyl inhibits Aβ deposition and Tau pathology in 3xTg mice.** (A) Representative Aβ PET images showing the effects of 11a, 11a-isopropyl, and NP-11a-isopropyl treatment on the Aβ plaque deposition in the brains of 3xTg mice. (B) Relative quantification of Aβ PET Standardized Uptake Value (SUV) in (A) (n ​= ​3 biological replicates, ∗p ​< ​0.05, compared with vehicle). (C) Immunofluorescence staining of Aβ in the hippocampus of 3xTg mice with chronic treatment. Scale bar, 20 ​μm. (D) Relative quantification of Aβ fluorescence intensity in (C) (20 positive cells from three mice for statistics, ∗p ​< ​0.05, ∗∗p ​< ​0.01, compared with vehicle). (E) ELISA analysis of mouse Aβ42 (left panel) and Aβ40 (right panel) levels in brain tissue of 3xTg mice with chronic treatment. (n ​= ​5 biological replicates, ∗p ​< ​0.05, compared with vehicle). (F) and (G) SIMOA analysis of AEP downstream targets Tau N368 levels and p-Tau 181 in CSF (F) and plasma (G) of 3xTg mice with chronic treatment (n ​= ​5 biological replicates, ∗p ​< ​0.05, ∗∗p ​< ​0.01, and ∗∗∗*p* ​< ​0.001, compared with vehicle). (H) Representative Western blot images showing the effects of chronic treatment on the expression of AT8 and T22. (I) Relative quantification of AT8 and T22 levels in (H). T22 (aggregated Tau) and p-Tau AT8 levels showed reduction trends but did not reach statistical significance, potentially reflecting treatment duration limits in clearing established aggregates. (n ​= ​3 biological replicates, ∗p ​< ​0.05, ∗∗p ​< ​0.01, and ∗∗∗*p* ​< ​0.001, compared with vehicle).

Previous studies have shown that 3xTg mice develop detectable Aβ deposits in the brain around the age of 6 months. To monitor whether these drugs reduce Aβ plaques, we conducted an Aβ PET assay using ^18^F-AV45 one month after treatment. Quantification analysis revealed that SUVR values exhibited a reduction trend after these drug treatments, with 11a-isopropyl significantly decreasing the PET signals (Fig. 6A and B). IF co-staining with the hippocampal sections using anti-Aβ (6E10) demonstrated that Aβ aggregates in the brain were largely removed after the drug treatment (Fig. 6C and D). Quantification with Aβ ELISA indicated that Aβ42 levels were significantly reduced by 11a-isopropyl, while the other two drugs showed a reduction trend that did not reach statistical significance (Fig. 6E). Remarkably, quantification of Tau N368 and p-Tau 181 concentrations using Single Molecular Array (SIMOA) in 3xTg mice demonstrated that these markers were significantly decreased in both CSF and plasma after drug treatment (Fig. 6F and G). However, immunoblotting showed that T22, a biomarker for aggregated Tau, and p-Tau AT8 levels were not significantly reduced by these treatments (Fig. 6H and I). This suggests that the duration of pharmacological intervention may have been insufficient to remove aggregated Tau in 3xTg mice effectively. Behavioral tests revealed that treatment with 11a, 11a-isopropyl, and NP-11a-isopropyl significantly improved cognitive function in the Y maze but not in the novel objective recognition (Fig. S3). This further supports the notion that the treatment period may have been too short to alleviate Tau pathology in 3xTg mice.

## Discussion

In this study, we replaced the N-methyl group on 11a with an isopropyl group to synthesize 11a-isopropyl, aiming to stabilize the compound and enhance its hydrophobicity, thereby increasing brain exposure. We previously demonstrated that the N-methyl group in 11a is metabolically labile, as evidenced by liver microsomal assays and *in vivo* metabolite studies (Table S1). The isopropyl modification improved metabolic stability and increased permeability, as confirmed by the PAMPA-BBB assay (from 1.62 to 8.4; Table S1). Although the *in vitro* AEP inhibitory potency decreased (IC_50_: 6.8 ​nM for 11a vs. 19.2 ​nM for 11a-isopropyl), the compound retained comparable cellular efficacy in primary neurons (IC_50_: 45–63 ​nM), robustly blocking APP N585 and Tau N368 cleavage in a dose-dependent manner (Fig. 1). To further improve oral bioavailability and brain penetration, we encapsulated 11a-isopropyl in zein/LF NPs. This formulation enhanced oral bioavailability from 67.16 ​% to approximately 100 ​%, and significantly increased C_max_ in the brain, from 27.012 ​ng/mL (11a at 10 ​mg/kg) [37] to 321.248 ​ng/mL (11a-isopropyl at 5 ​mg/kg), reaching 561.002 ​ng/mL with NP delivery (Fig. 3). These improvements highlight the success of formulation strategies in elevating systemic and brain exposure.

However, critical pharmacokinetic limitations must be acknowledged. K_puu_ was estimated under the idealized assumption of no brain tissue binding by combining the measured brain-to-plasma ratio (C_max_, _brain_/C_max_, _plasma_ ​= ​0.067, Fig. 3F) with the unbound plasma fraction (f_u, plasma_ ​= ​0.204, Table S1). This yields an upper-bound K_puu_ of 0.328, which marginally exceeds the CNS drug threshold of 0.3 (estimated, not directly measured) [38]. The unbound brain concentration of NP-11a-isopropyl reached 28.1 ​ng/g (conservatively assuming the f_u,brain_ ≈ 0.05) [38,39], which exceeds the estimated target engagement threshold of ≈ 7.5 ​ng/g (based on IC_50_ ​= ​19.2 ​nM). The low K_puu_ suggests poor intrinsic BBB permeability, likely due to P-gp-mediated efflux liability (Table S1), although Caco-2 BCRP assays indicated that 11a-isopropyl is not a BCRP substrate (Table S2). Despite these challenges, NP-11a-isopropyl demonstrated improved PK/PD relationships and superior *in vivo* efficacy relative to the parent compound. In the 3xTg AD mouse model, NP-11a-isopropyl reduced AEP activation and downstream cleavage of APP and Tau, accompanied by decreased Aβ42 and p-Tau (AT8) levels (Fig. 4, Fig. 5, Fig. 6). These molecular changes correlated with partial restoration of cognitive function (Fig. S3A), supporting the therapeutic potential of the compound.

The zein-based NP platform was pivotal to this improvement. Zein, the primary storage protein in maize, constitutes approximately 45–50 ​% of the total protein in corn. It is amphiphilic, with a high content of hydrophobic amino acids, rendering it water-insoluble [29]. Consequently, zein-based delivery systems, such as films and nanoparticles, exhibit hydrophobic properties that enable controlled release of encapsulated compounds. These systems are biodegradable and can non-specifically carry a wide range of compounds, including curcumin, resveratrol, folic acid, atorvastatin, and daidzin, significantly enhancing their oral bioavailability [[40], [41], [42], [43], [44]]. The zein/LF nano-encapsulation strategy likely mitigated P-gp-mediated efflux at the intestinal epithelium and BBB, improving brain penetration (Fig. 3), consistent with prior findings that surface-modified NPs can bypass efflux transporters [25,26].

While surface engineering enhances nanoparticle efficacy, its implementation requires rigorous optimization, as demonstrated by both literature precedents and our systematic screening. For example, Lei et al. developed a “nano-cleaner” featuring a reactive oxygen species (ROS)-responsive PLGA core loaded with rapamycin and surface-functionalized with the KLVFF peptide and an acid-cleavable DAG peptide. This system accelerates Aβ degradation while reducing oxidative stress and inflammatory responses [45]. After evaluating various mass ratios of 11a-isopropyl and zein, a 1:10 ratio was selected based on parameters such as particle size, PDI, turbidity, EE(%), and LC(%). LF, known for its ability to stabilize NPs, also facilitates absorption via transferrin receptors on intestinal epithelial cells. LF-stabilized zein NPs show improved stability across a wide pH range and high salt concentrations. A 1:1 zein-to-LF ratio was identified as optimal based on PDI, particle size, turbidity, EE(%), and LC(%) (Fig. S1). Consequently, 11a-isopropyl/zein/LF NPs were prioritized for further *in vivo* PK/PD studies. While this strategy improved the compound's pharmacokinetic profile, limitations remain.

The incomplete reversal of Tau pathology highlights these limitations. Although SIMOA assays showed reduced CSF and plasma Tau N368 and pTau181, immunoblotting revealed no significant reduction in T22 or AT8 in the cortex (Fig. 6). This suggests that extracellular Tau was more readily cleared than intraneuronal aggregates. In 3xTg mice, Tau pathology becomes noticeable in the brain starting from 9 to 12 months old [46]. The drug intervention duration from 5 to 6 months of age in 3xTg mice, may have been sufficient to eliminate extracellular secreted Tau, including Tau N368 and p-Tau 181. However, this duration might have been too short to effectively reduce intra-neuronally deposited Tau aggregates, resulting in suboptimal attenuation of Tau pathology and only partial restoration of cognitive functions (Fig. 6H and I, Fig. S3). Moreover, modest improvement in the Novel Object Recognition Test (Fig. S3) and limited changes in aggregated Tau may reflect a combination of factors: subtherapeutic free brain drug levels, compensatory protease activity (e.g., caspase-2 or calpain) [47,48], and inter-individual variability within a small cohort (n ​= ​6). While a downward trend in AT8 and T22 was noted (Fig. 6H), the current sample size likely lacked power to detect subtle effects. To address these shortcomings, future work should incorporate larger cohorts, extended treatment durations, and dual-target approaches that address both Aβ and Tau, as well as metabolic and inflammatory contributors to AD.

Our comparative ADMET analysis further contextualizes these findings. While 11a exhibited better liver microsomal and hepatocyte stability in rodents, both compounds were similarly stable in human systems. 11a-isopropyl had significantly higher permeability (Pe ​= ​8.4 ​× ​10^−6^ ​cm/s vs. 1.62 ​× ​10^−6^ ​cm/s for 11a), reflected in higher AUCs in both plasma (4050.458 vs. 516.028) and brain (385.358 vs. 128.980) following oral administration (Fig. 3F) [37]. These values were further enhanced by NP formulation (AUC_0-t_: 6046.24 plasma; 425.02 brain) (Fig. 3A–F). In summary, zein/LF nanoencapsulation effectively enhanced the oral bioavailability and brain exposure of 11a-isopropyl. However, intrinsic pharmacokinetic barriers, including insufficient unbound brain concentrations and modest efficacy on intracellular Tau aggregates, underscore the need for further optimization. Future work should aim to refine both inhibitor design and delivery systems to achieve durable, brain-penetrant AEP inhibition. Such dual-optimization strategies will be crucial to fully realize the therapeutic potential of AEP-targeted interventions in AD.

## Material and methods

### Mice and reagents

CD-1 (ICR) mice were purchased from Beijing Vital River Laboratory Animal Technology Co., Ltd. (Beijing, China), and 3xTg mice were originally sourced from The Jackson Laboratory. All animals were housed and bred under specific pathogen-free (SPF) conditions at the Shenzhen Institute of Advanced Technology (SIAT), Chinese Academy of Sciences. Mice were maintained on a 12-h light/dark cycle with ad libitum access to food and water. Both male and female mice were used interchangeably in all experimental procedures.

### Primary neuron cultures

Primary rat cortical neurons were cultured as previously described. On day 13 *in vitro* (DIV13), neurons were treated with varying concentrations of 11a-isopropyl (0, 5, 10, 50, 100, 500, or 1000 ​nM) or pretreated with 11a-isopropyl or vehicle for 30 ​min before Aβ42 exposure. Subsequently, neurons were incubated with 2 ​μM pre-aggregated Aβ42 for 24 ​h.

### *In vivo* pharmacokinetics (PK) analysis

For *in vivo* pharmacokinetic (PK) studies, 8-week-old male CD-1 (ICR) mice were administered 11a-isopropyl via intravenous injection at a dose of 2 ​mg/kg (dissolved in saline) or via oral gavage at a dose of 5 ​mg/kg, either as a solution in 5 ​% DMSO/0.5 ​% methylcellulose or formulated as 11a-isopropyl/zein/LF nanoparticles (NP-11a-isopropyl). Blood and brain tissues were collected at 0, 0.08, 0.25, 0.5, 1, 2, 4, and 8 ​h post-administration. For acute pharmacodynamic (PD) assessments, male and female 3xTg mice received oral doses of NP-11a-isopropyl at 7.5 ​mg/kg or 15 ​mg/kg, and blood and brain samples were collected 2 ​h after treatment. In the efficacy study, 5-month-old male and female 3xTg mice were randomly assigned to four groups (n ​= ​8 biological replicates per group) and orally treated once daily for one month with vehicle, #11a (7.5 ​mg/kg), 11a-isopropyl (7.5 ​mg/kg), or NP-11a-isopropyl (7.5 ​mg/kg).

All compounds used were of high purity (>98 ​%) as confirmed by HPLC. 11a and 11a-isopropyl (≥98 ​%) were synthesized by Sundia Meditech Co. Ltd. (Shanghai, China). Lactoferrin (≥98 ​%) was obtained from Glycarbo Co., Ltd. (Tokyo, Japan). zein (≥95 ​%) and dextran (40 ​kDa) were purchased from Sigma-Aldrich (Missouri, USA). HPLC-grade methanol and ethanol were obtained from TEDIA (Ohio, USA). All other reagents were of analytical grade and sourced from Sinopharm Chemical Reagent Co. (Shanghai, China).

### Fabrication of zein, zein/LF, and zein/DLF NPs

Zein and zein/lactoferrin (LF) nanoparticles (NPs) were prepared based on previously reported methods, with slight modifications using an anti-solvent co-precipitation (ASCP) technique. Briefly, zein powder was accurately weighed and dissolved in 80 ​% aqueous ethanol to a final concentration of 1 ​% (w/v). The zein–ethanol solution was added dropwise to an LF aqueous solution at a 1:3 ​vol ratio using syringes under continuous stirring at 800 ​rpm for 30 ​min at room temperature. Deionized water served as the antisolvent to facilitate NP formation. Ethanol was subsequently removed via rotary evaporation at 40 ​°C under reduced pressure. The resulting nanoparticle dispersion was stored at 4 ​°C, and aliquots were freeze-dried for further use.

### Preparation of 11a-isopropyl-loaded zein, zein/LF, and zein/DLF NPs

11a-isopropyl-loaded zein nanoparticles (zein/11a-isopropyl) were prepared following the same procedure used for the fabrication of zein, zein/LF, and zein/DLF nanoparticles. Zein and 11a-isopropyl were co-dissolved in 80 ​% (v/v) ethanol at mass ratios of 5:1, 10:1, or 20:1. For the preparation of 11a-isopropyl–loaded zein/LF and zein/DLF nanoparticles (11a-isopropyl/zein/LF and 11a-isopropyl/zein/DLF), a mass ratio of 1:1 (zein to LF) was used. All formulations were stored at 4 ​°C, and portions were freeze-dried for subsequent analyses.

### Particle size, PDI, zeta potential

Particle size, polydispersity index (PDI), and zeta potential of the nanoparticles were measured using dynamic light scattering (DLS) with a Nano-ZS 90 instrument (Malvern Instruments Ltd., Worcestershire, UK). The turbidity of freshly prepared dispersions was evaluated by measuring absorbance at 600 ​nm using a microplate reader (BioTek Synergy HTX Multimode Reader, Agilent Technologies, California, USA) at room temperature.

### Entrapment efficiency and drug loading capacity determination

Nanoparticles (NPs) were centrifuged at 10,000×*g* for 10 ​min at 4 ​°C to separate unencapsulated 11a-isopropyl (#2). The supernatant containing encapsulated 11a-isopropyl was collected and diluted fivefold with methanol. To determine total drug content, an equal volume of the initial nanoparticle suspension was dissolved in methanol and similarly diluted. Entrapment efficiency (EE%) and loading capacity (LC%) were calculated using the following formulas:

EE(%)=(encapsulated11a−isopropyl/initial11a−isopropyl)×100%

LC(%)=(encapsulated11a−isopropyl/weightofunloadedcarrier)×100%

Quantification of encapsulated and total 11a-isopropyl was performed using a high-performance liquid chromatography (HPLC) system (LC-20A series, Shimadzu Corporation, Kyoto, Japan) equipped with a dual-solvent manager, injection system, column heater, and diode array detector (DAD). Chromatographic separation was carried out on a Phenomenex Luna C18 column (5 ​μm, 250 ​× ​4.6 ​mm; Phenomenex Ltd., Washington, USA).

### Morphological studies of the nanoparticles

#### Transmission electron microscopy (TEM)

Nanoparticle (NP) dispersions were diluted 10-fold with distilled water, deposited onto formvar–carbon-coated copper grids, and allowed to air-dry. The samples were stained with 2 ​% uranyl acetate, and excess stain was gently removed using filter paper. TEM imaging was performed using an HT7700 transmission electron microscope (HITACHI, Tokyo, Japan) operated at an acceleration voltage of 80 ​kV.

#### Scanning electron microscopy (SEM)

For SEM analysis, NP suspensions were freeze-dried and subsequently coated with a thin layer of gold using a sputter coater under vacuum to prevent surface charging. The surface morphology of the freeze-dried nanoparticles was observed using a field emission scanning electron microscope (APREO S, Thermo Fisher Scientific, Massachusetts, USA) at an accelerating voltage of 5 ​kV.

### SIMOA assay of CSF and blood samples

CSF and plasma samples were collected as previously described [49]. All Single Molecule Array (SIMOA) assays were performed using the Quanterix SR-X analyzer (Quanterix, USA). Human phosphorylated Tau at threonine 181 (p-Tau181) levels were quantified using the Simoa® pTau-181 Advantage V2 Kit (catalog #103714), according to the manufacturer's protocol. CSF and plasma samples were diluted 1:100 and 1:6, respectively, for these assays. Tau N368 levels were measured using a custom SIMOA assay developed with the Quanterix homebrew protocol. In this assay, monoclonal antibodies specific to Tau N368 were conjugated to paramagnetic beads, and the corresponding detection antibodies were biotinylated. Following optimization, the assay was run on the Quanterix SR-X analyzer. CSF and plasma samples were diluted at 1:400 and 1:20, respectively. All assays were conducted in a single experimental batch using the same reagent lots to ensure consistency and reduce inter-assay variability.

### ELISA assay

Mouse brain tissue samples were homogenized in ice-cold lysis buffer and centrifuged at 16,000×*g* for 20 ​min at 4 ​°C to separate soluble and insoluble protein fractions. The soluble supernatant was collected and quantified for target proteins using commercial ELISA kits, including human Aβ40 and Aβ42, adhering to the manufacturer's standardized protocols.

### Small animal PET

Mice were maintained under isoflurane inhalation anesthesia during all procedures to ensure stable physiological conditions for radiochemical administration, imaging, and data processing. PET imaging was conducted using a high-resolution small-animal scanner (microPET; spatial resolution: 1.0 ​mm). Following intravenous administration of 10–15 MBq of ^18^F-florbetapir (AV45) in 100 ​μL saline via the tail vein, 20-min volumetric brain emission scans were acquired. Image reconstruction was performed using ordered-subset expectation maximization (OSEM) to optimize spatial resolution and signal-to-noise ratios. For quantitative analysis, standardized uptake value ratios (SUVRs) were calculated by normalizing 18F-AV45 uptake in target regions to the cerebellar reference tissue. A predefined volumetric region of interest (VOI) template was applied to ensure consistent anatomical localization across subjects. SUVR data were statistically analyzed using Amide 1.0.4–1 (San Diego, CA, USA), with results reported for cortical and subcortical regions implicated in amyloid pathology.

### Novel object recognition test

Two identical cube-shaped objects (3 ​× ​3 ​× ​3 ​cm) were positioned in diagonally opposite corners of a square open-field arena (40 ​× ​40 ​cm), each placed 5 ​cm from the adjacent walls. Mice were habituated to the arena and objects during a 5-min baseline session. After a 1-h retention interval, one cube was substituted with a novel sphere-shaped object of comparable volumetric dimensions (3 ​cm diameter) to introduce a shape-based novelty. The same mouse was then reintroduced to the arena for a 5-min test session, during which exploratory behavior was recorded via an overhead camera. Locomotion and object interaction parameters (e.g., time spent exploring each object, proximity duration) were quantified using EthoVision XT 12 software (Noldus, Wageningen, The Netherlands).

### Y-maze test

The Y-maze test was employed to evaluate spatial working memory and spontaneous alternation behavior, a measure of natural exploratory pattern integrity. The apparatus comprised three symmetrical opaque arms (A, B, C; 30 ​cm length ​× ​8 ​cm width ​× ​15 ​cm height) intersecting at 120° angles to form a Y-shaped configuration. Mice were habituated to the maze during a 5-min acclimatization period to minimize novelty-induced anxiety. Following habituation, spontaneous exploration was recorded for 8 ​min using an overhead camera, with all trials conducted under consistent low-light conditions to reduce environmental stress. EthoVision XT 12 software (Noldus, Wageningen, The Netherlands) quantified total arm entries, entry sequences, and alternation patterns (consecutive entries into three distinct arms without repetition). The maze was thoroughly cleaned with 70 ​% ethanol between trials to eliminate olfactory cues.

### AEP activity assay

AEP activity was assessed as previously described [21]. Tissue homogenates (10 ​μg protein) were incubated with 20 ​μM AEP-specific fluorogenic substrate in 200 ​μL of optimized assay buffer (20 ​mM citric acid, 60 ​mM Na_2_HPO_4_, 1 ​mM EDTA, 0.1 ​% CHAPS, 1 ​mM DTT; pH 6.0) to quantify enzymatic activity. Reactions were performed at 37 ​°C in a temperature-controlled microplate reader (BioTek Synergy HTX Multimode Reader, Agilent, California, USA). AEP-mediated cleavage of the substrate, liberating fluorescent 7-amino-4-methylcoumarin (AMC), was monitored kinetically by measuring emission at 460 ​nm (excitation: 380 ​nm) every 5 ​min over 60-min. Fluorescence values were normalized to background signal from substrate-free controls, and enzyme activity was calculated as the rate of AMC generation (RFU/min/μg protein) during the linear phase of the reaction.

### Western blot analysis

Mouse brain tissue or cultured cells were homogenized in ice-cold lysis buffer (50 ​mM Tris-HCl, 40 ​mM NaCl, 1 ​mM EDTA, 0.5 ​% Triton X-100, 1.5 ​mM Na_3_VO_4_, 50 ​mM NaF, 10 ​mM sodium β-glycerophosphate/pyrophosphate, pH 7.4) with protease/phosphatase inhibitors. Lysates were incubated on ice (30 ​min), centrifuged (12,000×*g*, 20 ​min, 4 ​°C), and supernatants denatured in SDS-PAGE buffer (95 ​°C, 5 ​min). Proteins were resolved on 10 ​% SDS-PAGE gels, transferred to PVDF membranes, and blocked with 5 ​% non-fat milk in TBST (1 ​h, RT). Membranes were incubated with primary antibodies (4 ​°C, overnight), washed, treated with HRP-conjugated secondary antibodies (1 ​h, RT), and visualized using ECL Prime. Band intensity was quantified via Image J.

### Immunofluorescence staining

Frozen tissue sections were subjected to antigen retrieval by immersion in 10 ​mM citric acid buffer (pH 6.0), heated to boiling for 15 ​min, and subsequently cooled on ice. Following retrieval, sections were treated with a permeabilization buffer (containing 0.1 ​% Triton X-100; Beyotime Biotechnology, Beijing, China) for 15 ​min. After three washes with 1 ​× ​PBST, non-specific binding was blocked using 1 ​% bovine serum albumin (BSA) for 1 ​h at room temperature. Primary antibodies were applied to the sections and incubated overnight at 4 ​°C. Unbound antibodies were removed via three PBST washes, after which sections were incubated with a multiplexed mixture of Alexa Fluor 488-, 555-, and 647-conjugated secondary antibodies (Invitrogen, California, USA) for fluorescent detection. Imaging was performed using a ZEISS LSM 980 confocal microscope (ZEISS Microscopy, Oberkochen, Germany), with consistent acquisition settings across samples.

## Statistical analysis

Data from a minimum of three independent biological replicates are expressed as mean ​± ​standard deviation (S.D.). For comparisons between two experimental groups, statistical significance was assessed using an unpaired two-tailed Student's t-test. Analyses involving three or more groups were performed via one-way ANOVA. A p-value <0.05 was considered statistically significant for all tests. All statistical procedures, including graph generation, were executed using GraphPad Prism software (version 9.0; GraphPad Software Inc., San Diego, CA, USA).

## Author contributions

Keqiang Ye conceived the project, designed the experiments, analyzed the data, and wrote the manuscript. Xin Meng and Mengmeng Wang performed most of the experiments. Seong Su Kang performed Fig. 1. Bowei Li and Wen-Hua Zheng assisted with data analysis and interpretation and critically read the manuscript. All of the authors are involved in analyzing the data and contributed to manuscript discussion and editing.

## Declaration of competing interest

The authors declare no competing financial interest.

## Figures and Tables

**figs1:**
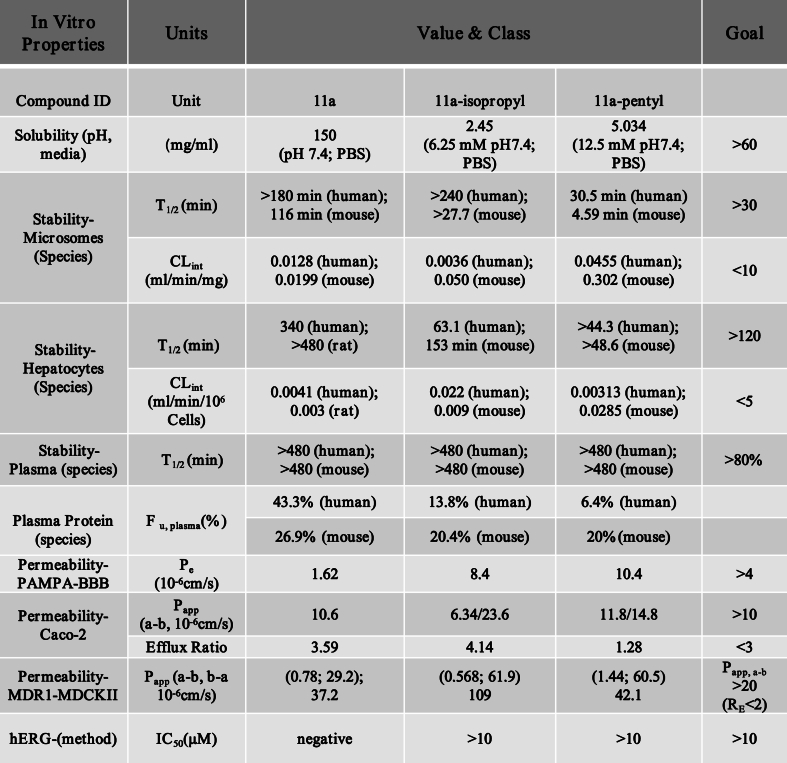


**figs2:**
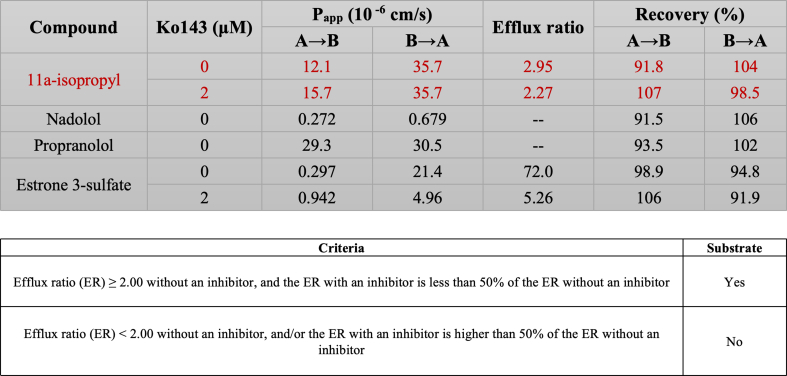


**figs3:**
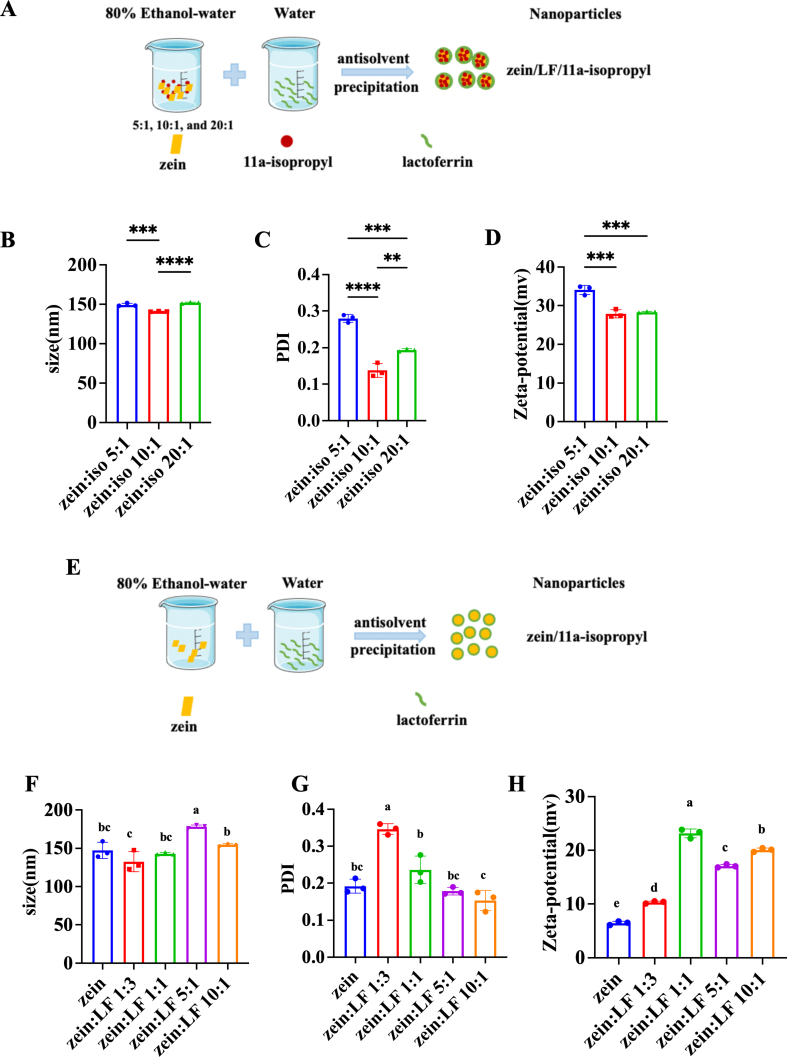


**figs4:**
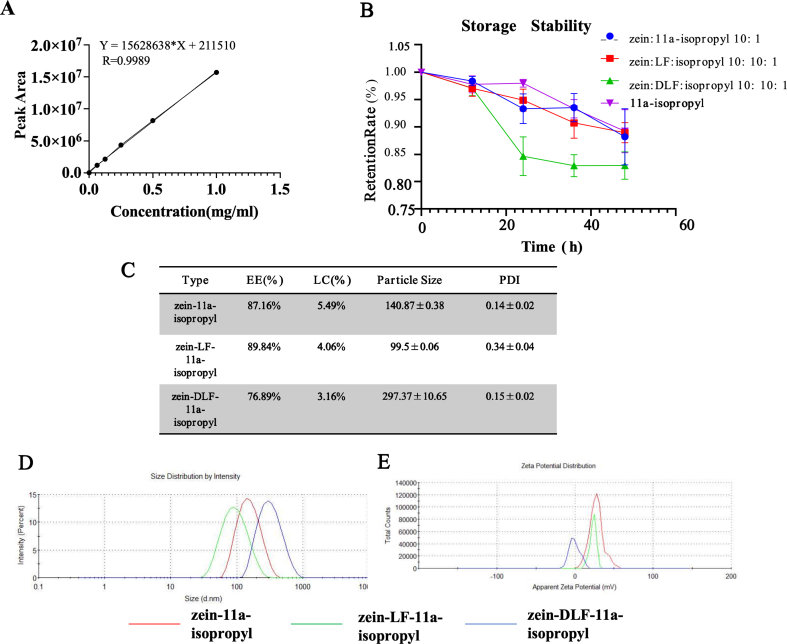


**figs5:**
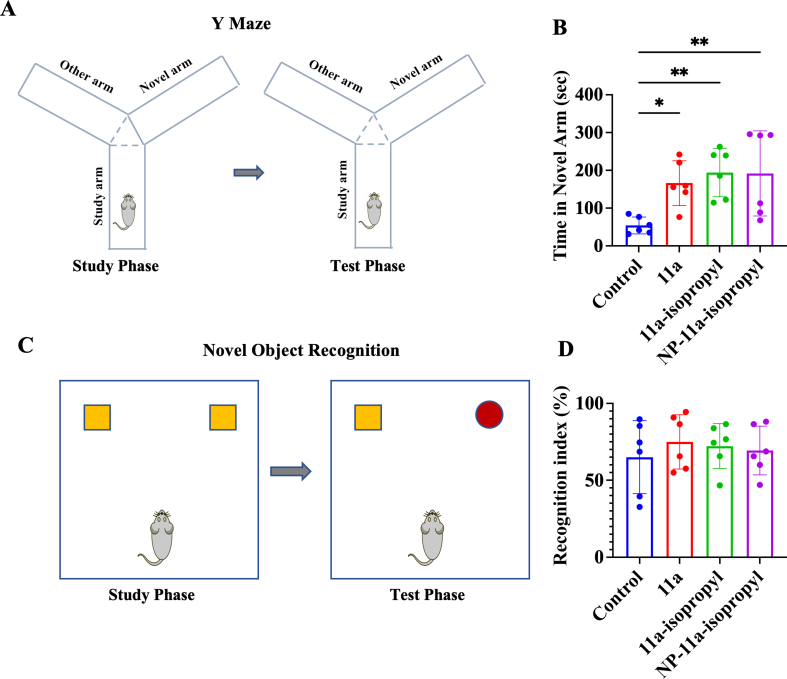


## References

[1] Glenner G.G., Wong C.W. (1984). Alzheimer's disease: initial report of the purification and characterization of a novel cerebrovascular amyloid protein. Biochem Biophys Res Commun.

[2] Grundke-Iqbal I., Iqbal K., Tung Y.C., Quinlan M., Wisniewski H.M., Binder L.I. (1986). Abnormal phosphorylation of the microtubule-associated protein tau (tau) in Alzheimer cytoskeletal pathology. Proc Natl Acad Sci USA.

[3] Dall E., Brandstetter H. (2016). Structure and function of legumain in health and disease. Biochimie.

[4] Xiong J., Zhang Z., Ye K. (2023). C/EBPβ/AEP signaling drives Alzheimer's disease pathogenesis. Neurosci Bull.

[5] Liu Z., Jang S.W., Liu X., Cheng D., Peng J., Yepes M. (2008). Neuroprotective actions of PIKE-L by inhibition of SET proteolytic degradation by asparagine endopeptidase. Mol Cell.

[6] Simonsen A.H., McGuire J., Podust V.N., Hagnelius N.O., Nilsson T.K., Kapaki E. (2007). A novel panel of cerebrospinal fluid biomarkers for the differential diagnosis of Alzheimer's disease versus normal aging and frontotemporal dementia. Dement Geriatr Cogn Disord.

[7] Yates C.M., Butterworth J., Tennant M.C., Gordon A. (1990). Enzyme activities in relation to pH and lactate in postmortem brain in Alzheimer-type and other dementias. J Neurochem.

[8] Fang B., Wang D., Huang M., Yu G., Li H. (2010). Hypothesis on the relationship between the change in intracellular pH and incidence of sporadic Alzheimer's disease or vascular dementia. Int J Neurosci.

[9] Mathews P.M., Levy E. (2016). Cystatin C in aging and in Alzheimer's disease. Ageing Res Rev.

[10] Zhang Z., Song M., Liu X., Su Kang S., Duong D.M., Seyfried N.T. (2015). Delta-secretase cleaves amyloid precursor protein and regulates the pathogenesis in Alzheimer's disease. Nat Commun.

[11] Quinn J.P., Corbett N.J., Kellett K.A.B., Hooper N.M. (2018). Tau proteolysis in the pathogenesis of tauopathies: neurotoxic fragments and novel biomarkers. J Alzheimers Dis.

[12] Zhao X., Kotilinek L.A., Smith B., Hlynialuk C., Zahs K., Ramsden M. (2016). Caspase-2 cleavage of tau reversibly impairs memory. Nat Med.

[13] de Calignon A., Fox L.M., Pitstick R., Carlson G.A., Bacskai B.J., Spires-Jones T.L. (2010). Caspase activation precedes and leads to tangles. Nature.

[14] Ferreira A., Bigio E.H. (2011). Calpain-mediated tau cleavage: a mechanism leading to neurodegeneration shared by multiple tauopathies. Mol Med.

[15] Kang S.S., Ahn E.H., Ye K. (2020). Delta-secretase cleavage of Tau mediates its pathology and propagation in Alzheimer's disease. Exp Mol Med.

[16] Zhang Z., Tian Y., Ye K. (2020). δ-secretase in neurodegenerative diseases: mechanisms, regulators and therapeutic opportunities. Transl Neurodegener.

[17] Basurto-Islas G., Grundke-Iqbal I., Tung Y.C., Liu F., Iqbal K. (2013). Activation of asparaginyl endopeptidase leads to Tau hyperphosphorylation in Alzheimer disease. J Biol Chem.

[18] Zhang Z., Song M., Liu X., Kang S.S., Kwon I.S., Duong D.M. (2014). Cleavage of tau by asparagine endopeptidase mediates the neurofibrillary pathology in Alzheimer's disease. Nat Med.

[19] Xia Y., Wang Z.H., Zhang Z., Liu X., Yu S.P., Wang J.Z. (2021). Delta- and beta- secretases crosstalk amplifies the amyloidogenic pathway in Alzheimer's disease. Prog Neurobiol.

[20] Zhang Z., Li X.G., Wang Z.H., Song M., Yu S.P., Kang S.S. (2021). δ-secretase-cleaved Tau stimulates Aβ production via upregulating STAT1-BACE1 signaling in Alzheimer's disease. Mol Psychiatry.

[21] Zhang Z., Obianyo O., Dall E., Du Y., Fu H., Liu X. (2017). Inhibition of delta-secretase improves cognitive functions in mouse models of Alzheimer's disease. Nat Commun.

[22] Yao M., Xiao H., McClements D.J. (2014). Delivery of lipophilic bioactives: assembly, disassembly, and reassembly of lipid nanoparticles. Annu Rev Food Sci Technol.

[23] Pouton C.W., Porter C.J. (2008). Formulation of lipid-based delivery systems for oral administration: materials, methods and strategies. Adv Drug Deliv Rev.

[24] McClements D.J. (2010). Emulsion design to improve the delivery of functional lipophilic components. Annu Rev Food Sci Technol.

[25] Ruan S., Zhou Y., Jiang X., Gao H. (2021). Rethinking CRITID procedure of brain targeting drug delivery: circulation, blood brain barrier recognition, intracellular transport, diseased cell targeting, internalization, and drug release. Adv Sci (Weinh).

[26] Wohlfart S., Gelperina S., Kreuter J. (2012). Transport of drugs across the blood-brain barrier by nanoparticles. J Control Release.

[27] Liu G., An D., Li J., Deng S. (2023). Zein-based nanoparticles: preparation, characterization, and pharmaceutical application. Front Pharmacol.

[28] Raza A., Shen N., Li J., Chen Y., Wang J.Y. (2019). Formulation of zein based compression coated floating tablets for enhanced gastric retention and tunable drug release. Eur J Pharm Sci.

[29] Paliwal R., Palakurthi S. (2014). Zein in controlled drug delivery and tissue engineering. J Control Release.

[30] Wang Y., Padua G.W. (2012). Nanoscale characterization of zein self-assembly. Langmuir.

[31] Patel A.R., Bouwens E.C., Velikov K.P. (2010). Sodium caseinate stabilized zein colloidal particles. J Agric Food Chem.

[32] Chen Y., Zhao Z., Xia G., Xue F., Chen C., Zhang Y. (2020). Fabrication and characterization of zein/lactoferrin composite nanoparticles for encapsulating 7,8-dihydroxyflavone: enhancement of stability, water solubility and bioaccessibility. Int J Biol Macromol.

[33] Ou A.T., Zhang J.X., Fang Y.F., Wang R., Tang X.P., Zhao P.F. (2021). Disulfiram-loaded lactoferrin nanoparticles for treating inflammatory diseases. Acta Pharmacol Sin.

[34] Suzuki Y.A., Lopez V., Lönnerdal B. (2005). Mammalian lactoferrin receptors: structure and function. Cell Mol Life Sci.

[35] Wang G., Han J., Meng X., Kang S.S., Liu X., Sun Y.E. (2023). Zein-based nanoparticles improve the therapeutic efficacy of a TrkB agonist toward Alzheimer's disease. ACS Chem Neurosci.

[36] Liao J., Chen C., Ahn E.H., Liu X., Li H., Edgington-Mitchell L.E. (2021). Targeting both BDNF/TrkB pathway and delta-secretase for treating Alzheimer's disease. Neuropharmacology.

[37] Qian Z., Li B., Meng X., Liao J., Wang G., Li Y. (2024). Inhibition of asparagine endopeptidase (AEP) effectively treats sporadic Alzheimer's disease in mice. Neuropsychopharmacology.

[38] Kalvass J.C., Maurer T.S., Pollack G.M. (2007). Use of plasma and brain unbound fractions to assess the extent of brain distribution of 34 drugs: comparison of unbound concentration ratios to in vivo p-glycoprotein efflux ratios. Drug Metab Dispos.

[39] Hammarlund-Udenaes M., Fridén M., Syvänen S., Gupta A. (2008). On the rate and extent of drug delivery to the brain. Pharm Res.

[40] Brotons-Canto A., González-Navarro C.J., Gil A.G., Asin-Prieto E., Saiz M.J., Llabrés J.M. (2021). Zein nanoparticles improve the oral bioavailability of curcumin in Wistar rats. Pharmaceutics.

[41] Nunes R., Baião A., Monteiro D., das Neves J., Sarmento B. (2020). Zein nanoparticles as low-cost, safe, and effective carriers to improve the oral bioavailability of resveratrol. Drug Deliv Transl Res.

[42] Peñalva R., Morales J., González-Navarro C.J., Larrañeta E., Quincoces G., Peñuelas I. (2018). Increased oral bioavailability of resveratrol by its encapsulation in Casein nanoparticles. Int J Mol Sci.

[43] Hashem F.M., Al-Sawahli M.M., Nasr M., Ahmed O.A. (2015). Optimized zein nanospheres for improved oral bioavailability of atorvastatin. Int J Nanomed.

[44] Zou T., Gu L. (2013). TPGS emulsified zein nanoparticles enhanced oral bioavailability of daidzin: in vitro characteristics and in vivo performance. Mol Pharm.

[45] Lei T., Yang Z., Xia X., Chen Y., Yang X., Xie R. (2021). A nanocleaner specifically penetrates the blood‒brain barrier at lesions to clean toxic proteins and regulate inflammation in Alzheimer's disease. Acta Pharm Sin B.

[46] Oddo S., Caccamo A., Shepherd J.D., Murphy M.P., Golde T.E., Kayed R. (2003). Triple-transgenic model of Alzheimer's disease with plaques and tangles: intracellular Abeta and synaptic dysfunction. Neuron.

[47] Chen H.H., Liu P., Auger P., Lee S.H., Adolfsson O., Rey-Bellet L. (2018). Calpain-mediated tau fragmentation is altered in Alzheimer's disease progression. Sci Rep.

[48] Yang J., Shen N., Shen J., Yang Y., Li H.L. (2024). Complicated role of post-translational modification and protease-cleaved fragments of tau in Alzheimer's disease and other tauopathies. Mol Neurobiol.

[49] Qian Z., Li H., Yang H., Yang Q., Lu Z., Wang L. (2021). Osteocalcin attenuates oligodendrocyte differentiation and myelination via GPR37 signaling in the mouse brain. Sci Adv.

